# Unraveling the Mechanisms of Cutaneous Graft-Versus-Host Disease

**DOI:** 10.3389/fimmu.2018.00963

**Published:** 2018-05-02

**Authors:** Pedro Santos e Sousa, Clare L. Bennett, Ronjon Chakraverty

**Affiliations:** ^1^UCL Cancer Institute, University College London, London, United Kingdom; ^2^UCL Institute of Immunity and Transplantation, University College London, London, United Kingdom

**Keywords:** cutaneous graft-versus-host disease, pathophysiology, T cells, antigen-presenting cells, B cells, repair mechanisms, microbiome

## Abstract

The skin is the most common target organ affected by graft-versus-host disease (GVHD), with severity and response to therapy representing important predictors of patient survival. Although many of the initiating events in GVHD pathogenesis have been defined, less is known about why treatment resistance occurs or why there is often a permanent failure to restore tissue homeostasis. Emerging data suggest that the unique immune microenvironment in the skin is responsible for defining location- and context-specific mechanisms of injury that are distinct from those involved in other target organs. In this review, we address recent advances in our understanding of GVHD biology in the skin and outline the new research themes that will ultimately enable design of precision therapies.

## Introduction

Graft-versus-host disease (GVHD) is a severe immune-related complication of allogeneic hematopoietic stem cell transplantation (allo-HSCT), affecting 30–50% of transplanted patients and associated with both treatment resistance and excess mortality (1). Recognized by Billingham as a syndrome in which donor T cells recognize and attack host tissues in an immunocompromised recipient (2), GVHD has a wide range of clinical manifestations involving predominantly the skin, gastrointestinal tract, liver, and lungs. Although GVHD has been classically divided into acute or chronic subtypes depending on the time of its clinical onset, it is now accepted that the kinetics of acute and chronic GVHD are variable and that disease features frequently overlap (3). It is, therefore, not clear if acute and chronic GVHD are indeed sequential phases of the same disease or two independent disorders, with unique molecular and pathophysiological mechanisms (4–7). Although biomarkers have been identified that can predict acute and chronic GVHD onset and severity (8–14), it is unclear whether earlier therapeutic interventions in patients predicted to develop severe GVHD can change its natural history.

The skin is the most frequently affected organ both in acute and chronic GVHD (15, 16), and the cutaneous manifestations are often the presenting sign of the disease (17); the extent of involvement correlates with prognosis either by acting as a surrogate for GVHD severity at other sites (18), or more directly, by leading to loss of essential barrier functions and infections. In its acute form, the most prominent skin features include the sudden development of a symmetrical maculopapular rash, predominantly on the upper back and neck, the palms/soles, and face, which may progress to a diffuse erythroderma with the formation of bullae and epidermal necrosis (19). Histologically, cutaneous acute GVHD is characterized by an interface dermatitis, with extensive leukocyte infiltration of the superficial dermis and vacuolization of the epidermal basal layer (20). Apoptosis of individual keratinocytes along the basement membrane, including a subset of cytokeratin 15-expressing putative stem cells is a characteristic feature of acute injury (21). In contrast, cutaneous chronic GVHD may present a wide range of manifestations reflecting a spectrum of epidermal (lichenoid) and dermal (sclerodermatous) changes (22). Lichen planus-like eruptions and poikiloderma affecting the dorsal aspects of the hands, forearms, and trunk are the most typical features of early stage non-sclerotic cutaneous chronic GVHD, its histopathological hallmarks being hyperkeratosis, focal hypergranulosis, acanthosis, basal cell necrosis, vacuolar degeneration of the basal layer, and a distinctive superficial perivascular or band-like lymphoid cell infiltrate with perifollicular fibrosis (20). Sclerodermatous lesions usually develop progressively over the trunk, buttocks, hips, and thighs, with localized morphea-like features, diffuse sclerosis, or lichen sclerosus-like features, characterized by collagen homogenization of the dermis and/or subcutaneous tissues (17, 20). In this mini-review, we will address the pathophysiology of cutaneous GVHD as addressed mostly using models of experimental allo-HSCT in mice, highlighting recent advances in the field and discussing some of the key challenges that remain.

## Overview of GVHD Pathogenesis

The pathogenesis of systemic acute and chronic GVHD has been extensively characterized in preclinical models and the reader is referred to several, excellent reviews (23–27). Briefly, initiation of acute GVHD is triggered by tissue injury as a result of the preparatory conditioning regimens leading to increased exposure to damage- or pathogen-associated molecules that activate host antigen-presenting cells (APC) (28). Host APC activation primes the proliferation of alloantigen-specific donor T cells and their migration toward target sites leading to immune-mediated injury through a broad array of cytotoxic and cytokine dependent mechanisms, a process that is amplified by the recruitment of additional effector populations. It is increasingly recognized that distortions to the local gut microbiome exacerbate injury by further reducing epithelial integrity at a time when repair mechanisms are also perturbed (28, 29). The mechanisms underlying chronic GVHD are multifaceted and include thymic injury and disordered T cell selection, loss of regulatory populations, disruption to B cell homeostasis, and aberrant tissue repair with fibrosis (30).

**Figure 1:**
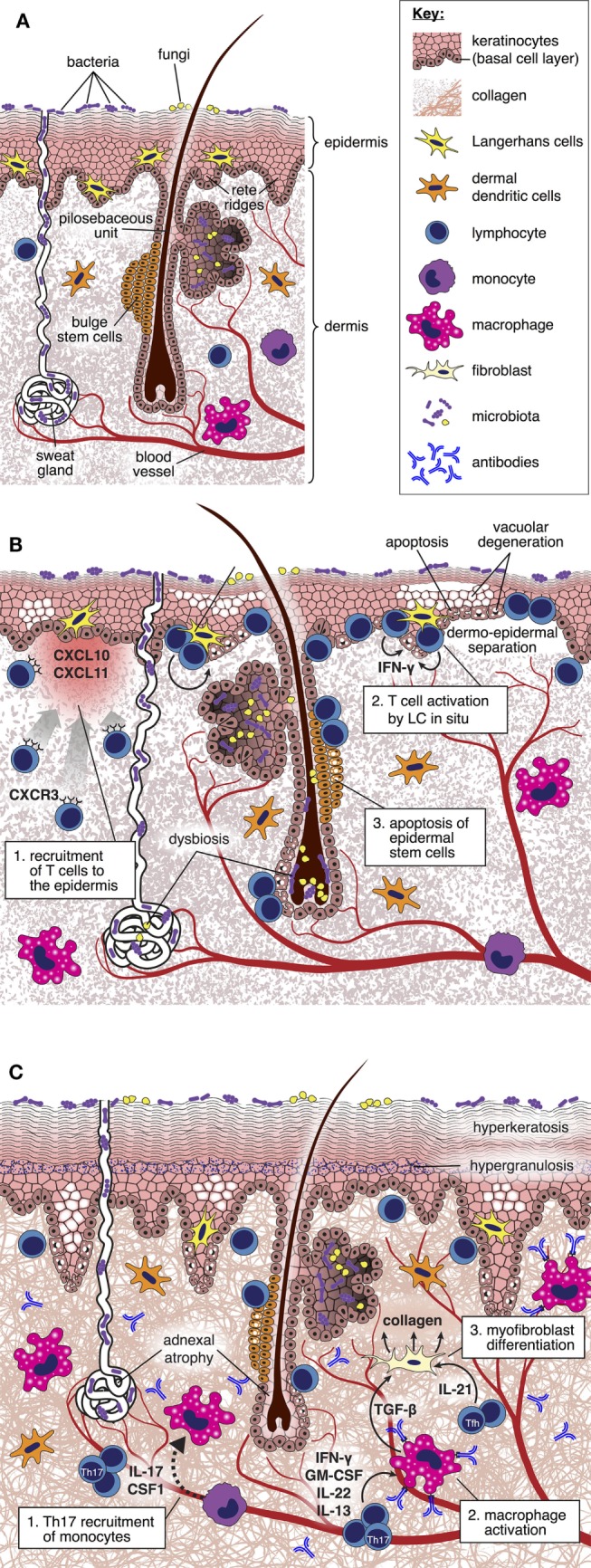
Pathogenesis of cutaneous graft-versus-host disease (GVHD). **(A)** Diagram depicting cellular and structural elements of normal skin. **(B)** During acute GVHD, the local production of IFN-γ inducible chemokines in response to tissue injury promotes the recruitment of CXCR3^+^ alloreactive T cells into the skin (34). Upon *in situ* interaction with host-type Langerhans cells, alloreactive T cells are reprogramed to differentiate into pathogenic effector cells characterized by enhanced survival and generation of IFN-γ (35). Pathogenic T cells kill Lgr5^+^ epidermal stem cells (36), thus disabling normal repair mechanisms and epidermopoiesis; these effects are manifested by vacuolar degeneration and apoptosis of the basal and suprabasal epidermal cells, focal dermo-epidermal separation, and necrosis of the epidermis with denudation. Distortion of the local microbiome as a consequence of the conditioning regimen and antibiotic therapy may also be an important factor driving skin inflammation (37). **(C)** During the evolution of chronic GVHD, donor T cell-derived IL-17 is required for recruitment of Ly6C^low^ monocytes to the skin, which then differentiate into macrophages (38). Generation of transforming growth factor (TGF)-β by activated macrophages induces fibroblasts to differentiate into myofibroblasts that promote collagen deposition through a heat shock protein 47-dependent mechanism (39). Binding of allo- or auto-antibodies to macrophage Fc receptors induces their polarization toward the M2 phenotype and production of TGF-β (27), thus amplifying the process of myofibroblast generation of collagen. IL-21 generation by follicular helper-like cells recruited to the skin may also have direct effects upon skin fibroblast differentiation (40). Collectively, these mechanisms lead to thickening and homogenization of collagen bundles throughout the dermis.

Although the skin is the major site of involvement, only a minority of experimental studies focus specifically upon distinct mechanisms that underpin injury at this location. Analysis of the kinetics of gene expression associated with the development of cutaneous GVHD in rodents has suggested complex spatial and temporal transcriptional signatures linked to inflammation, antigen presentation, effector cell recruitment and activation, apoptosis, and tissue repair, indicative of a sequential infiltration and activation of effector cells that correlate with *in situ* morphological alterations (31–33). Assembling this information into a comprehensive framework explaining the pathogenesis of skin GVHD and suggesting novel, targetable pathways remains a considerable challenge. Below, we review the existing literature as it relates to cutaneous GVHD and indicate those areas where we believe that critical information is still lacking (Figure 1).

## Pathogenic Mechanisms in Skin GVHD

### The Role of T Cells

An extensive summary of the role of individual T cell subsets and cytokines in GVHD pathogenesis is beyond the scope of this discussion, but we refer readers to recent reviews by experts in the field (27, 41). In murine experimental systems, no single T cell differentiation pathway can explain the full spectrum of tissue injury at all sites; plasticity and heterogeneity of early T cell effector programs (35, 42), variation in housing conditions, clinical relevance of the mouse models employed (43), and the potential impact of immune suppressants that are used in the clinic (44) all complicate this issue further. In addition, experiments using genetic approaches to block differentiation down one pathway may distort the overall balance of differentiation across other subsets, making the results often difficult to interpret (45). Finally, cytokine profiling of human T cells derived from skin lesions from patients with acute or chronic GVHD reveal a greater level of heterogeneity than suggested by the animal models, cautioning against underestimating the complexity of this disorder (34, 46).

Proliferative, Th1 cell responses were long considered to represent the paradigm for T cell differentiation in acute GVHD (47, 48); in experimental BMT, donor T cell responsiveness to the Th1-related cytokine, IFN-γ, is in turn required for CXCR3 expression and recruitment to the skin, suggesting a feed-forward loop that drives injury (34). The requirement of Th1 differentiation in driving acute GVHD has been challenged by numerous studies, which have demonstrated that Th2 or Th17 polarized cells can also induce acute skin injury in mice (45, 49–51). For example, a skewing toward Th2/Tc2 differentiation in *Stat4^−/−^* splenocyte donors increases acute GVHD of the skin (52). *In vitro* polarized Th17 cells also induce greater acute GVHD of the skin when transferred to allo-HSCT recipient mice (50), akin to their pathogenic role in other autoimmune and skin inflammatory diseases, such as psoriasis (53), scleroderma (54), and lichen planus (55). In a murine model of acute GVHD where we tracked the evolution of effector T cell gene expression according to time and location, we recently showed that transcriptional profiles of CD8^+^ effector T cells in the epidermis were highly distinct from other GVHD sites (including the dermis) and did not readily conform to previously published Tc1 or Tc17 signatures (35). Taken together, these data suggest that there is substantial redundancy in the requirements for acute skin injury in GVHD and/or that the precise molecular mechanisms leading to pathogenicity do not segregate exclusively with the “classic” differentiation pathways. Reciprocal to enhancement of effector T cell expansion, acute GVHD is also associated with an early deficiency in Treg cells, a process that contributes to skin injury (56). In a human skin explant model that mimics acute GVHD, Treg cells block the priming of CD8^+^ effector T cells (57) and their subsequent homing to skin by reducing T cell expression of CXCR3 and skin-expression of CXCL10/CXCL11 (58).

Loss of Treg cells during experimental acute GVHD may also be permissive for the subsequent emergence of pathogenic autoreactive donor T cells capable of inducing chronic tissue damage (59, 60). Consistent with this concept, therapeutic use of low dose IL-2 in human patients with chronic GVHD enhances Treg expansion and is associated with significant improvements in skin disease (61). In human allo-HSCT patients, chronic lichenoid GVHD is characterized by a mixed Th1/Th17 signature, a profile that is distinct from that observed in early lesions where Th2 and IL-22-producing T cells were found to predominate (46). Interestingly, while recipient-derived IL-17 and IL-22 are protective against acute GVHD in mice, preventing gut dysbiosis and helping maintaining epithelial integrity in the gastrointestinal tract (62, 63), dysregulated production of the same cytokines by donor-derived T cells drive pathogenicity in chronic skin GVHD, leading to abnormal differentiation of keratinocytes and dermal fibrosis (44, 51). Skin-infiltration by ICOS^+^ follicular helper (Tfh)-like CD4^+^ T cells and ICOS^+^ Th1 cells may also be important in driving skin fibrosis in murine chronic GVHD, the latter process requiring the generation of the Tfh-related cytokine, IL-21 (40); of note, antibody-mediated depletion of ICOS-expressing T cells or IL-21 blockade was able to block collagen deposition. Like acute GVHD, the human and murine studies in chronic GVHD are indicative of substantial heterogeneity of T cells recruited to the skin; a priority, therefore, will be to define pathways that are both pathogenic and targetable.

### The Role of APCs

Although host-derived hematopoietic APC have been proposed to act as central players in acute GVHD initiation (64, 65), further studies using stringent conditional ablation of individual BM-derived APC subsets in mice have failed to show an absolute requirement of any single population for the induction of systemic disease (24); indeed, there appears to be considerable redundancy in the requirement for professional APC, especially, for CD4^+^ T cell-dependent models of GVHD where non-hematopoietic cells are sufficient to induce widespread tissue injury (66).

An area under active investigation is the role of tissue APC populations, particularly, within target organs affected by the disease. Through their functions as sensors capable of integrating complex environmental cues, resident, or recruited APC are well placed to influence effector T cell functions as they enter peripheral tissues. In the context of infection, cognate interactions with monocyte-derived CD11c^+^ dendritic cells recruited to inflamed tissues are necessary for amplification of local cytokine generation and proliferation of activated T cells in mice (67, 68). It is possible that this process becomes corrupted during the development of GVHD, a concept that is supported by the involvement of other CD11c^+^ APC populations recruited to the sites of T-cell mediated immunopathology in autoimmune (69–71) or infection-related (72) inflammation. In the skin, the role of epidermal Langerhans cells (LCs) has received significant attention because this population is radio-resistant and host-derived LC can persist long term following allo-HSCT in human patients especially following transplantation involving T cell depletion or reduced intensity conditioning (73). In an MHC-mismatched model of murine allo-HSCT, host LCs were shown to be capable of inducing acute skin GVHD (74), thus overriding their steady-state role in promoting tolerance (75). However, whether host LC populations are required for initiation of acute GVHD under conditions where other APC populations can efficiently present host antigens is controversial. In human patients, the persistence of recipient-derived LC does not predict clinical or histological skin GVHD (76), although this does not exclude a role of this population is initiating inflammation. Using models of inducible depletion in mice, we have found that host LC can “instruct” local T cell pathogenicity in an MHC-mismatched model of allo-HSCT where local GVHD was triggered by local application of a toll-like receptor agonist (77) and in several independent models of MHC-matched, minor antigen mismatched allo-HSCT (35). Host LCs were not required for activation of CD8^+^ T cells within the draining lymph node or subsequent homing of effector cells to the epidermis. Instead, epidermal LC triggered pathogenic differentiation of T cells *in situ*, an effect that was linked to the expression of a pro-inflammatory gene cluster that was also conserved in human patients at the onset of acute GVHD (35). In contrast, another study using Langerin.DTA BMT recipients (a model in which recipients lack LC through their lifespan) found that skin GVHD was unaffected in the absence of LC (78). However, these latter experimental findings are difficult to interpret because long-term absence of LC may be associated with abnormal baseline immunity and exaggerated T cell immunity (79). The finding that keratinocytes overexpressing a self-antigen can also activate naive CD8^+^ T cells directly and induce a local GVHD-like inflammation (80) suggests the possibility that allogeneic T cells entering the skin can potentially be regulated by multiple cell types *via* cognate interactions; in this case, the outcome of the interaction could be specific to the T cell clone or to the antigen involved.

Recruitment of other myeloid populations to the skin may also have the potential to influence local immunity. In models of acute systemic GVHD, early persistence of host-derived macrophages can limit GVHD through a process involving the CD47-dependent engulfment of allo-reactive T cells (81). Similarly, in collaboration with the Rubio group, we have found that granulocyte-colony stimulating factor (G-CSF)-mobilized peripheral blood stem cell grafts (which are used routinely in clinical practice) contain high frequencies of a variant, immunosuppressive CD34^+^ mature monocyte population that inhibits allogeneic T cell proliferation and correlates with a reduced risk of GVHD (82). A similar variant Ly6C^high^ monocyte population can be found in the spleens of G-CSF-treated mice (82, 83); upon activation with IFN-γ, these cells produce NO that inhibits T cell function and also prevents GVHD. In contrast, emerging data support the concept that donor-derived monocytes and macrophages can also amplify GVHD. In human patients with GVHD, intermediate CD14^++^CD16^+^ monocytes can promote the induction of a subset of Th17 cells that are resistant to glucocorticoids (38). Furthermore, several clinical studies have reported a strong correlation between cutaneous macrophage infiltration and acute and chronic GVHD, in particular, in cases associated with steroid resistance and poor prognosis (84). In preclinical models of Th17-dependent chronic sclerodermatous GVHD, donor-derived M2-like macrophages infiltrate the skin in a macrophage colony-stimulating factor (CSF)-1 and CSF-1 receptor-dependent manner and contribute to dermal sclerosis *via* a mechanism that requires transforming growth factor (TGF)-β (85, 86). A recent study in mice has shown that TGF-β drives the differentiation of fibroblasts into collagen-producing myofibroblasts that express the collagen molecular chaperone, heat shock protein 47 (HSP47); of note, knock down of HSP47 can significantly block the process of collagen deposition in chronic GVHD (39). The immediate precursor population that traffics to the skin and gives rise to the macrophage population is not known, but appears to be distinct from Ly6C^high^ inflammatory-type monocytes, which are usually thought to perform this role. Like LC, a sub-population of CD1a^−^ CD14^+^ Factor XIIIa^+^-resident dermal macrophages are resistant to the conditioning therapy, retaining a mixed host–donor chimerism for over 1 year following allo-HSCT (87); *ex vivo*, this population is capable of regulating CD4^+^ T cell cytokine generation and promoting memory CD8^+^ T cell proliferation, suggesting their potential to sustain the alloreactive response (87).

### The Role of B Cells and Antibodies

Profound disruption to B cell homeostasis is seen in chronic GVHD in human patients, with reduced reconstitution of memory B cells or IL-10 producing regulatory B cells and reciprocal increases in activated transitional B cells (88–90). B-cell supporting structures (e.g., the germinal center), cytokines (e.g., B-cell activating factor), and accessory populations (e.g., Tfh and regulatory populations) are also affected by chronic GVHD in murine studies. Although not required to initiate tissue injury, donor-derived B cell antibodies deposit in murine skin, where they promote cutaneous Th17 infiltration, leading to perpetuation of local chronic GVHD (91). In humans with chronic GVHD, auto-antibodies to platelet-derived growth factor induce accumulation of reactive oxygen species (ROS), and stimulate type 1 collagen gene expression through the Ha-Ras-ERK1/2-ROS signaling pathway (92); this process may be important in initiating the pathological fibrotic response. These findings provide the rationale for targeting B cells using Rituximab or using drugs that interfere with B-cell signaling to prevent or treat GVHD, the latter approach being associated with promising early results (93).

### The Role of Repair Mechanisms

Ongoing inflammation and/or damage to normal repair processes lead to a profound failure to restore normal skin tissue homeostasis in untreated GVHD. In models of acute GVHD, early tissue responses including new blood vessel (94) and lymphatic formation (95) are abnormal even before the influx of T cells. Furthermore, the normal restoration of epithelial integrity through proliferation and differentiation of adjacent epidermal stem cells within the hair follicle or inter-follicular epithelium does not occur (96). Instead, the epidermal proliferative response is uncoupled from differentiation (97) leading to dysregulation of epidermopoiesis. This abnormal differentiation program leads to the induction of genes that are not expressed by keratinocytes in the normal skin and the abnormal overexpression of differentiation markers (31), in a similar manner to that described in other inflammatory skin disorders, such as psoriasis and atopic dermatitis (98, 99). Experimental and human studies in acute GVHD have shown that apoptotic cells in the epidermis are spatially restricted to sites that harbor putative epidermal stem cells in the hair follicle bulge or human rete ridges, or rete-like prominences (RLP) found in the murine dorsal tongue (100–103). A recent study by the Teshima group has demonstrated that leucine-rich repeat-containing G-protein coupled receptor (Lgr)5^+^ epidermal stem cells in the RLP of the murine dorsal tongue and bulge region of hair follicles are targeted early following the initiation of GVHD (36). Loss of epidermal stem cells in GVHD led to both impairment in the normal progression to hair follicle growth (anagen) and a reduced capacity for wound healing (36). Of note, use of topical corticosteroids compounded loss of Lgr5^+^ cells in the epithelium, whereas inhibition of JAK1/2 with Ruxolitinib was effective at blocking T cell infiltration and restoring Lgr5^+^ cell populations. It is, therefore, likely that the disordered attempt at repair in GVHD relates significantly to the targeting of stem cells necessary to restore epithelial integrity. The role of other factors relevant to disordered repair requires further investigation but could include examining the impact of the inflammatory microenvironment upon basement membrane integrity, the constitution of the extracellular matrix, the availability of local differentiation signals, and the effect upon accessory populations.

### The Role of the Skin Microbiome

A large number of recent studies have detailed the changes in the gut microbiome during the development of acute GVHD in human patients (29), where intestinal injury is associated with loss of diversity and increases in pathogenic bacteria. In murine models, intestinal dysbiosis in GVHD can disrupt epithelial integrity by interfering with the formation of mucus or the intraluminal generation of short chain fatty acids that fortify gap junctions (104–107). Other changes in the human gut virome during GVHD are also emerging (108). To date, the impact of GVHD upon the skin microbiota and its potential role in promoting injury has not been defined. In the steady state, the human skin microbiome is highly stable (with *Propionobacterium, Corynebacterium, Staphylococcus*, and *Melassezia* being the most prominent genera) and possesses a significantly smaller biomass than the gut (109). Relative acidity and the presence of antimicrobial molecules, such as free fatty acids and antimicrobial peptides that inhibit microbial colonization, make conditions unfavorable for the growth of certain microorganisms (109). Conditioning, the use of antibiotics, inflammation, and relative immune deficiency all have the potential to distort the local microbiota. Skin-resident microorganisms have been shown to control the expression of different antimicrobial peptides, such as cathelicidins and β-defensins (110), as well as components of the complement system (111) while enhancing the induction and/or activation of lymphocytes both in the steady state and during infection (112, 113). In a murine model of atopic dermatitis, for instance, it has been found that dysbiosis is an important pathological factor driving skin inflammation, with the emergence of *Staphylococcus aureus* and *Corynebacterium bovis* as dominant populations being correlated with the induction of dermatitis and the enhancement of Th2 responses, respectively, in a process mediated by LC (114). The association between alterations in the diversity and composition of the skin microbiota has now been reported in a variety of other inflammatory skin conditions; however, for most of them, it is not yet clear whether these are the cause or a consequence of the disease (37). Studies in gut GVHD have provided an experimental framework for testing the role of specific changes to the microbiota in the skin and testing the feasibility of its manipulation therapeutically.

## Concluding Remarks

Precision-based therapies for skin GVHD will require an in depth understanding of tissue-specific processes that initiate or amplify disease. There remains a significant information gap in relation to the specific pathogenesis of skin GVHD because only a minority of preclinical studies focus primarily on injury to this site. The distinct immune microenvironment of the skin and the likely context-specific interactions between multiple cell types cautions against extrapolation of mechanisms pertinent to other sites involved by GVHD. Future research should also focus upon pathological immune responses as measured in tissues rather than secondary lymphoid organs, and refine models to include components that reflect the complexity of the clinical setting (for example, with the use of prophylactic immune suppression or antibiotics). Corroborative evidence that mechanisms identified in preclinical models are conserved in human patients will be essential in selection of potential targetable pathways. Ultimately, the next step change will derive from unbiased, systems-level approaches interrogating whole tissues to identify candidate mechanisms that drive inflammation.

## Author Contributions

PS, CB, and RC formulated opinions and concepts for the mini-review and wrote it together.

## Conflict of Interest Statement

The authors declare that the research was conducted in the absence of any commercial or financial relationships that could be construed as a potential conflict of interest.
